# Development of a dynamic water budget model for Abu Dhabi Emirate, UAE

**DOI:** 10.1371/journal.pone.0245140

**Published:** 2021-01-27

**Authors:** Mohamed I. Kizhisseri, Mohamed M. Mohamed, Walid El-Shorbagy, Rezaul Chowdhury, Adrian McDonald

**Affiliations:** 1 Civil and Environmental Engineering Department, College of Engineering, United Arab Emirates University, Al Ain, UAE; 2 National Water Center, United Arab Emirates University, Al Ain, UAE; 3 Civil and Architectural Engineering and Mechanics, University of Arizona, Tucson, Arizona, United States of America; 4 School of Civil Engineering and Surveying, University of Southern Queensland, Toowoomba, Australia; 5 Faculty of Environment, University of Leeds, Leeds, United Kingdom; Soil and Water Resources Institute ELGO-DIMITRA, GREECE

## Abstract

In this study, a dynamic water budget model is developed for the Emirate of Abu Dhabi (EAD) in the United Arab Emirates (UAE). The model, called Abu Dhabi Water Budget Model (ADWBM), accounts for a number of drivers such as population growth, economic growth, consumption pattern and climatic factors. Model formulation, calibration, validation as well as simulation results for two future situations are presented in this paper. The two water simulations discuss demand-side options in response to different future water conditions until 2050. The first simulation, namely, baseline (BL) simulation examined water balance in the emirate assuming no change in both water production and consumption. BL simulation results highlight the expected shortages in water resources assuming no modification in the supply side. The second simulation, a more conservative and practical simulation considering water conservation options and sustainable improvements to the supply side was developed to achieve a balanced water budget by reducing the baseline consumption rates. The results show that a significant demand reduction is needed in all demand sectors, reaching 60% in the potable sectors and above 70% in non-potable sectors. Overall, results show that the ADWBM can be used as a numerical tool to produce accurate figures of water supply and demand for the sake of planning and decision making in the water sector of the EAD until 2050.

## 1. Introduction

Water supply and demand is among the hot issues particularly in the countries with arid or semi-arid climatic conditions. The Middle East countries depend on desalinated water and groundwater, owing to its arid climate and limited precipitation, to meet its significant water needs. Desalinated water is the primary source of water for potable uses in the Middle East, while groundwater is extracted for agricultural and other irrigational use. Water demand in the Gulf Cooperation Council (GCC) countries has increased seven-fold in the last 40 years, from 5 Billion Cubic Meter / year (BCM/yr) to 35 BCM/yr, primarily as a result of population growth and accelerated socio-economic development [1]. The factors driving increased water consumption are population growth, economic development, and changes in lifestyle, which have increased the demand for water for irrigation, human consumption, and industrial processes. Agriculture accounts for the highest proportion of water use in the region [2]. Given the significant increase in water consumption in recent years, there is a strong effort by the governments to manage scarce water resources more sustainably. This calls for efficient management of water resources to address the future balance and imbalances between supply and demand. Thus, a mathematical tool that can assist the efficient management and conservation of water resources will be useful.

A water balance analysis of a hydrologic system in a region is essential for decision-making that involves management or planning of water resources. In order to conduct a water balance study, a mass balance analysis is required to be developed for the study area, including all the components of inflows, outflows, and storage within a defined boundary. The inflows are those components which adds to the water of a region while outflows refer to all water that are moving out the system. The storage component is change in the stored water calculated over a period. For any region, the major inflow are rainfall and surface inflows into the region through streams and rivers. The outflow components mainly consist of evapotranspiration from different land uses and flow of drains from the region to sea. Depending on the geographical location and climatic zone of a region, various spatial and temporal processes that affect the overall water balance of a hydrological system are different. In semi-arid and arid regions where the rainfall is scarce the major inflows are from the water taken up from sea into the terrestrial system. Semi-arid and arid climatic zones are also characterized by the evapotranspiration while the evaporation from the water bodies are negligible because surface water sources such as lakes and rivers are not present in such regions. Therefore, each of the water budget study carried at different geographical locations with specific purposes has developed their own methodologies suitable for their situation depending on the various water components present there. In arid regions, water resources are limited and thereby the available groundwater for irrigation and other water uses are severely constrained.

Several water balance models have been developed by researchers worldwide to solve numerous water-related issues at local, regional, and national levels. Various water balance reported across the world assess all water components in the study area [3–7]. Few attempts were made to outline water balancing approaches developed by various organizations, including the United Nations (UN), the International Water Management Institute (IWMI), and the Australian government [e.g. [8]].

The studies conducted in the arid and semi-arid regions focused on various climatic and hydrologic parameters that are distinct to desert conditions to analyze the surface and groundwater availability for various uses such as irrigational use, domestic use, and other nondomestic use by industrial and commercial sectors. In 2014, a study conducted in the semi-arid region located in Jordan used a transient model to examine the watershed for a mountainous region and found that evapotranspiration is the major component of the precipitation occurring there with a percentage of 87.5% [9]. A study by [10] analyzed the climatic water balance of a most arid region in Romania in which water dynamics of five decades were studied using data collected from nine weather stations, and deployed both statistical and GIS techniques for trend analysis at annual and seasonal scales. The study results showed that there is increase in water deficit over the last five decades and called for a more efficient management of water resources with more importance to be given for agricultural productivity. Water balance of arid regions in Columbia is analyzed under two climate change scenarios (RCP 4.5 and RCP 8.5) for two different time periods taking into consideration various parameters which are distinctive of an arid area such as evapotranspiration, soil moisture, recharge of the aquifer, runoff, water deficit and excess, and water use [11]. An estimation of the water balance in an arid region in Tanzania was done by [12] utilizing the remote sensing data. The spatial and temporal variability of water balance parameters within the catchment were studied to help lake management and conservation in relation to soil erosion, climate, and land use change. In a study conducted by [13] a dynamic water balance model was developed for key hydrological processes in drylands in Tunisia which is helpful for the spatial and temporal planning of water harvesting as well as in the optimization of agricultural activities. Roy and Duke Ophori (2012), in their study in semi-arid regions of California, analyzed the water balance to identify the seasonal variations in soil moisture, recharge, runoff to estimate the water surplus or deficit to judicious use of water for crop irrigation [14]. A quantification of precipitation, runoff, evapotranspiration, and drainage was done to understand the impact of climate and land use on hydrology of semi-arid savanna located in southwest United States [15]. In a study conducted in the driest part of Europe in south-east Spain used numerical models to test the various conceptual models and developed an improved water budget model for Torrevieja aquifer, thus helping in improved water management [16]. One of the major process that determines the available rainfall to support vegetation and recharge in an arid region is the surface evaporation. Some of the studies included the evapotranspiration estimation using SEC (Surface Evaporation Capacitor) model [17]. Yao et al. improved the measurement accuracy of evapotranspiration (ET), precipitation, and runoff estimates by merging remote sensing, reanalysis, data assimilation datasets, and ground observations [18]. In some studies, Satellite-based water cycle components such as precipitation from the Tropical Rainfall Measuring Mission (TRMM) and ET from the Moderate Resolution Imaging were used [e.g. [19]].

In the Middle East, UAE [20] has undertaken one of the few water budget studies conducted in the region. The study noted that the expected population growth will put additional stress on the water resources available in the country. There is, thus, a need for a water budget for the city, which the author presents in the paper. In a different study [21], focused on depletion of ground water resources and increased dependency on desalination due to population growth and economic development in UAE. They used the Gravity Recovery and Climate Experiment (GRACE) and TRMM data to understand the variations in groundwater storage as a balance of total rainfall, evapotranspiration, and desalinated water to help in the optimal allocation of water. From the literature review conducted, it is clear that for a sustainable water resources management, establishing the relationship between all water components is vital, but in addition employing analysis tools to simulate possible future scenarios are also required. Thus, once a water balance is established for any water system, it can be applied to develop a dynamic model which can forecast the future shifts. Dynamic modeling of water systems can be undertaken using a variety of approaches. System dynamics (SD) models and parameter models have been identified to be used in developing dynamic water budget models. There are dynamic models that use a set of validated parameters identified from water balance models to simulate future conditions. Jazim (2006) constructed a six-parameter water model to forecast monthly runoff at arid and semiarid catchments [22]. In Iran, Camp et al. (2015) deployed lumped parameter approach to develop a model that can be applied to intramountain basins [23]. Several summaries showing how various parameters used in selected models developed by different researchers have been previously presented (e.g. [24, 25]). Perez-Sanchez (2019) conducted a comparative study of six models developed in Spain during the period 1977–2010 to check the robustness of the methodology and concluded that all models considered in the study performed well in humid and sub-humid regions [26]. Other very recent studies illustrated the use of parametric approach for evaluating water supply security (e.g. [27, 28]).

Although there are numerous dynamic water budget models developed all over the world with specific purposes, to the best of our knowledge, a comprehensive dynamic model for long-term water scenarios development and analysis of future water situations is not available for any semi-arid or arid climatic region. Therefore, the focus of this study was to develop such a model to simulate future scenarios of the water system, which can thereby assist long-term planning and policy development for water budgeting of an arid region. In this research, a region in the Middle East, in the United Arab Emirates, namely the Emirate of Abu Dhabi is chosen for the study due to its arid climate similar to other Middle East countries. The specific objectives of this study are: 1) to construct a water balance model for EAD by collecting and analyzing all data relevant to water balance such as population growth, economic development, climate change and other physical conditions; 2) to identify key parameters and engage them to build a dynamic model for the EAD; 3) to calibrate and validate the model parameters using historical data followed by sensitivity analysis; 4) to develop future water simulations for a time horizon until the year 2050; 5) to predict water balances over time for the water simulations; and 6) to determine the conservation levels to be achieved for a balanced water budget (BWB) under each simulation.

## 2. Model development

### 2.1 Study area

**Fig 1:**
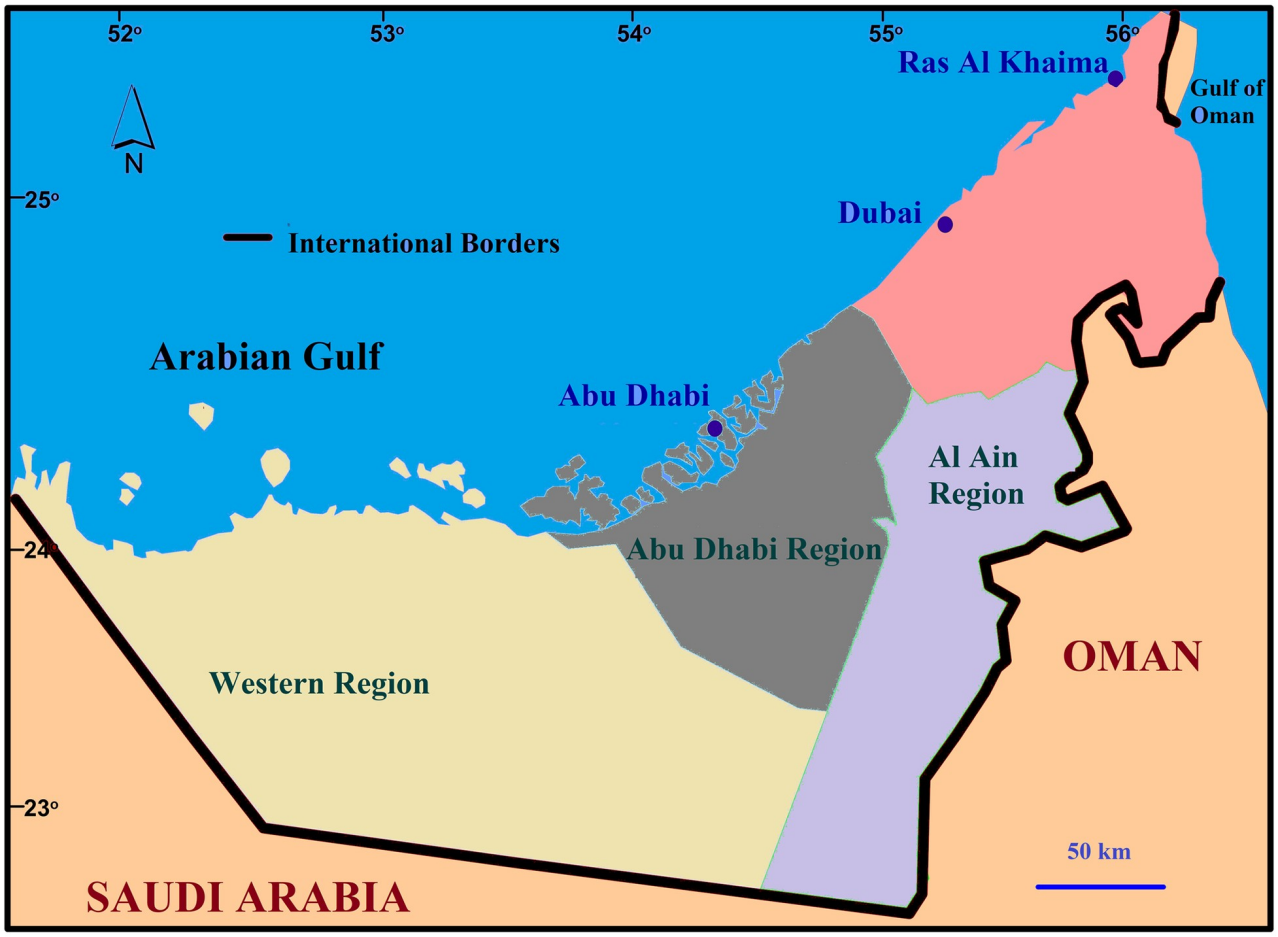
Proposed site of the study.

The proposed site of study is Abu Dhabi, the largest emirate of the seven emirates of UAE which covers an area of 67,340 km^2^, and is mostly a desert. It comprises three regions namely, Abu Dhabi, Al-Ain, and Western, as shown in Fig 1. The emirate shares its boundary with Oman in the east, Saudi Arabia to south and west, and the Arabian (Persian) Gulf in the north. The climatic condition is arid with a hot and humid atmosphere during the majority of the months. The maximum temperature averages above 40°C (104°F) during the April-September summer period. Abu Dhabi has a long coastline of more than 600 kms that produce humid climatic conditions due to the heat from the sun. The October-March period is comparatively cool. January and February are the coolest. As rainfall is rare, the natural recharge into groundwater is very low; it is about 40 MCM/yr [29], which adds to the water concerns of the emirate.

Over the last decades, water demand has increased significantly in the EAD, and the main driving forces are population growth and economic development. The population has increased multiple times in the past few decades and drives much of the water consumption increase in Abu Dhabi Emirate; especially, the residential, commercial, and municipal consumptions [30]. Since 1975, the total population has by more than a 6.6 times [30]. Moreover, changes in lifestyle have increased the water demand for irrigation, human consumption, and industrial activities. Several public policies intensified the increase in this water demand. Some of these policies encourage the expansion of agriculture to protect rural heritage and make the EAD less dependent on imported food. Some policies call for desert greening to provide a habitat for wild animals and stabilize the sand around roads. Some call for the development of public parks to enhance the aesthetic value of outdoor spaces, while others promote residential and commercial megaprojects to cater to the local population and the growing tourism industry. Industrialization is highly driven by the government’s diversification vision of conversion into non-oil industries.

### 2.2 Conceptual water balance model

**Fig 2:**
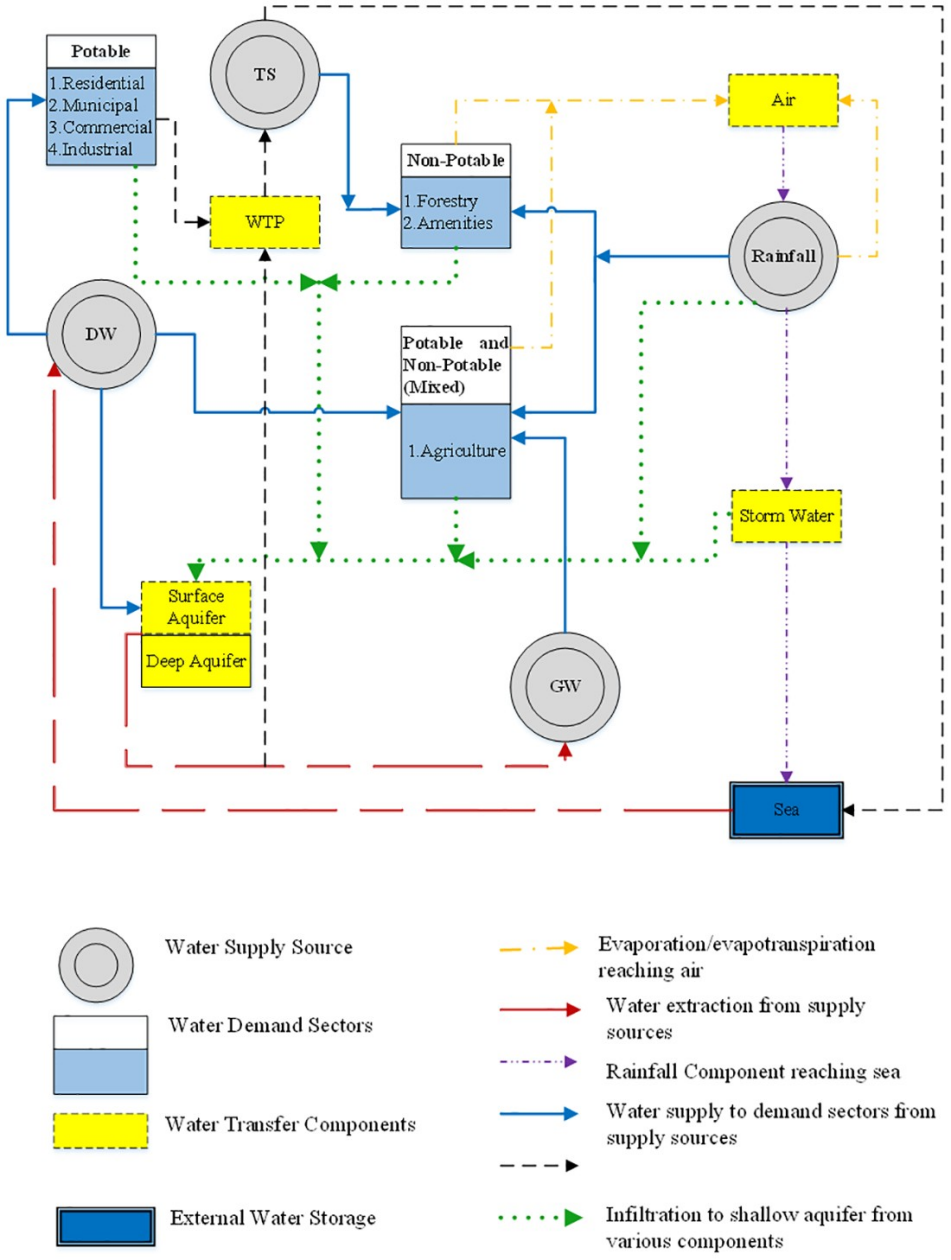
Schematic representation of water system of Abu Dhabi.

A systematic approach is applied to analyze the complex water system of the EAD aiming at developing a conceptual framework for the water balance model. The conceptual model was framed by investigating all the water inflows, outflows, and storage and transfer components, with the EAD chosen as the boundary for the study. A holistic water balance was established for the whole system. To develop the water balance model, the water system of Abu Dhabi was divided into three sub-systems: water supply subsystem, water demand subsystem, and water transfer subsystem. The conceptualized model structure of the system is shown in Fig 2. It showed that complex interactive relationships exist between the components of the three subsystems. All possible interlinks among the different water resources, demand sectors, and transfer constituents are considered in the model for developing the mass balance equations. Thus, a conceptual model that comprises four water supply sources, seven demand sectors, and three transfer components is developed for the EAD.

#### 2.2.1 Mass balance equations for water supply subsystem

This subsystem comprises the major water supply sources currently in use for meeting water demands in the EAD. In the model constructed, there are four water supply sources: groundwater (GW), desalinated water (DW), treated sewage (TS), and rainfall surface-runoff (RF_SR). The first three sources form the major contributors of water supply in Abu Dhabi. According to [31], about 62% of Abu Dhabi’s water supply is from GW, followed by 30.5% from DW, and 7.5% from TS. The mass balance equation for the total annual water supply is constructed as summation of the supplies from all these sources as (Eq 1):

$$WS_{Total}=\;GW_{Total}+\;DW_{Total}+\;TS_{Total}+\;RF\_SR_{Total},$$ (1)

where *WS*_*Total*_ is the total annual water supply, *GW*_*Total*_ is the total annual supply from GW, *DW*_*Total*_ is the total annual supply from *DW*, *TS*_*Total*_ is the total annual supply from TS, and *RF_SR*_*Total*_ is the total annual surface-runoff from the total rainfall (RF_*Total*_) received.

*Groundwater*. Groundwater is the largest supplier of water in the EAD. The groundwater in Abu Dhabi is either in shallow aquifers or deep aquifers, with shallow aquifers having the chances of rainfall recharges at a very low percent. To account for the flow of water from GW resource to various demand sectors, all the GW consumers were identified, and mass balance equation was formulated based on the GW extraction and consumption data. In the EAD, GW is used for irrigation in agricultural, forestry, and public amenities. This is represented by (Eq 2):

$$GW_{Total}=\;GW_{A}+\;GW_{AM}+\;GW_{F},$$ (2)

where *GW*_*A*_, *GW*_*AM*_, and *GW*_*F*_ are the annual groundwater consumption by agriculture, amenities (public spaces that include parks and open green area), and forestry, respectively. *GW*_*Total*_ is the total annual GW extracted.

*2*.*2*.*1*.*1 Desalinated water*. Desalinated water is the second-largest water supply source in Abu Dhabi. It is the main source of water for all potable demands; namely, residential, municipal, commercial, and industrial water uses. DW is also channeled to irrigation lands and groundwater recharge depending on surplus production. The production and consumption details of DW in the EAD are taken from the official website of Abu Dhabi Water and Electricity Company (ADWEC) [32]. Thus, the mass balance for DW produced and consumed was established as given by (Eq 3):

$$DW_{Total}=\;DW_{A}+\;DW_{F}+\;DW_{AM}+\;DW_{R}+\;DW_{M}+\;DW_{C}+\;DW_{I}+\;DW_{inf-SA}+DW_{AR-SA},$$ (3)

where *DW*_*A*_, *DW*_*F*_, *DW*_*AM*_, *DW*_*R*_, *DW*_*M*_, *DW*_*C*_, *and DW*_*I*_, are DW supplied to agriculture, forestry, amenities, residential, municipal, commercial, and industrial sectors, respectively. *DW*_*inf-SA*_ is the losses from transmission and distribution network leakages. *DW*_*AR-SA*_ is the DW used for artificial recharge of aquifers, implemented at the strategic locations in the EAD.

*2*.*2*.*1*.*2 Treated sewage*. TS refers to treated sewage and is considered to be an alternate source of water for non-potable uses. It is produced by treating the wastewater generated from residential, commercial, municipal, and industrial use to reusable quality, at the wastewater treatment plants (WTP). The EAD is using TS for irrigation of forestry and amenities. Even though the produced TS is of good quality enough for agriculture, due to acceptance barriers by the users, it is yet to be used for large scale agricultural irrigation. As a result, TS is supplied for forestry and amenities only. Currently, a portion of TS is discharged into the Arabian Gulf due to capacity limitations of the TS distribution system. The details of TS are collected from the Abu Dhabi Sewerage Services Company (ADSSC), [31, 33]. The TS balance was calculated considering all these components and is represented as in (Eq 4):

$$TS_{Total}=\;TS_{AM}+\;TS_{F}+\;TS_{Sea},$$ (4)

where *TS*_*Total*_ is the total annual TS produced, while *TS*_*AM*_ and TS_*F*_ represent TS supplied to amenities and forestry, respectively. TS_*Sea*_ is the portion of TS discharged into the sea.

*2*.*2*.*1*.*3 Rainfall*. Located in an arid region, the EAD receives very low rainfall; usually less than 100 mm per year [29]. Therefore, in the EAD, rainfall is an insignificant source of water supply. However, the mass balance of the rainfall was established by considering its various components and is given by (Eq 5).

$$RF\_SR_{Total}=\;RF_{Total}–\;RF_{SDS}-RF_{inf-SA}-\;RF_{E-OA},$$ (5)

where *RF_SR*_*Total*_ is the surface-runoff from rainfall that comprises *RF_SR*_*A*,_ *RF_SR*_*F*,_ and *RF_SR*_*AM*_ components that can be made available to irrigate agriculture, forestry, and amenities sectors, respectively. *RF*_*SDS*_ is the rainfall component which is discharged into the sea through storm water collection system present in Abu Dhabi. *RF*_*inf-SA*_ is the portion of rainfall that reaches shallow aquifer through infiltration. *RF*_*E-OA*_ is the evaporation components of rainfall received, which is lost into atmosphere.

#### 2.2.2 Mass balance equations for water demand subsystem

Water demand subsystem comprises all the water demand sectors, which are the consumers of water in the EAD. In the model, there are seven water demand sectors identified in the EAD: residential, municipal, commercial, industrial, amenities, agricultural, and forestry. All these demand sectors are classified as either potable or non- potable demand sectors based on the water resources it can depend on. Potable demand sectors are those which depend purely on the DW, the only source of potable water in the EAD, for their water needs. Non-potable demands sectors are those which can be depend on any of the non-potable sources, GW, TS or RF_SR.

*2*.*2*.*2*.*1 Residential*. Residential sector is a potable demand sector and is the largest consumer of DW in the EAD. Residential use comprises indoor use as well as outdoor use by the residents. The residential water consumption in the EAD is double the rate of many developed countries. This fact is mainly because of outdoor water uses, particularly for garden irrigation, and is influenced by subsidized water tariffs. Since DW is the only source of potable water, the residential consumption is given (Eq 6).

$${\text{R}}_{Consumption\_Total}=DW_{R},$$ (6)

where *R*_*Consumption_Total*_ is the total annual residential consumption and is supplied by DW.

*2*.*2*.*2*.*2 Municipal*. Municipal water demand is defined as the water demand by all the governmental offices and related institutions like embassy, ministry, police, cultural, mosques and so on. Municipal water demand is supplied with DW only, as in (Eq 7):

$${\text{M}}_{Consumption\_Total}=DW_{M},$$ (7)

where *M*_*Consumption_Total*_ is the total annual municipal consumption.

*2*.*2*.*2*.*3 Commercial*. The commercial sector mainly includes properties like hotels, restaurants, cafeterias, car washes, and laundries. The main source of water for this demand sector is DW, and this sector is the third-largest consumer of DW in the EAD. Commercial consumption is given by (Eq 8):

$${\text{C}}_{Consumption\_Total}=DW_{C},$$ (8)

where *C*_*Consumption_Total*_ is the total annual commercial consumption and is supplied by DW.

*2*.*2*.*2*.*4 Industrial*. Industrial demand refers to the water required for various industrial activities. The major industrial activities in EAD are oil and gas, petrochemical industries, construction, manufacturing and so on. These industries use water mainly for their process, cooling or washing. Very little information is available on industrial water consumption, and this made the analysis difficult. However, the amount of water used in the industrial sector is found to be very small and it accounts to about 2 per cent of the DW produced in the EAD [30, 31, 33]. Industrial consumption is given by (Eq 9):

$$I_{Consumption\_Total}=DW_{I},$$ (9)

where *I*_*Consumption_Total*_ is the total industrial consumption and is supplied by DW.

*2*.*2*.*2*.*5 Amenities*. Amenities sector includes the public parks, landscapes, gardens, recreational area, and roadside planting where the water is supplied as irrigation water. According to the estimation by [34], public realm amenities (including parks, gardens, recreational areas, and roadside planting) consumed about 10 per cent of the total water consumption. Amenities sector mainly depends on TS and GW. However, if DW is available, it is also supplied for amenities. The amenities consumption equation is developed as given by (Eq 10):

$$AM_{Consumption\_Total}=GW_{AM}+DW_{AM}+TS_{AM}+\;RF\_SR_{AM},$$ (10)

where *AM*_*Consumption_Total*_ is the total annual amenities consumption from all sources of water supply. The terms of RHS represent the water supply to amenities from GW, DW, TS, and surface-runoff, respectively.

*2*.*2*.*2*.*6 Agricultural*. Agriculture (A) is the largest water demand sector in the EAD. Agricultural demand is the irrigational water use in the cultivated area of the three main crop types: fruit trees, field crops, and vegetable crops. Agriculture demand is met by GW (non-potable) extraction and DW (potable) supply. There is no metered estimation of GW withdrawal for agricultural purposes. In the absence of exact metered data, the estimated values as reported by [29, 33] were used to establish the mass balance, as shown in (Eq 11):

$$A_{Consumption\_Total\;\;}=GW_{A}+DW_{A}+\;RF\_SR_{A},$$ (11)

where *A*_*Consumption_Total*_ is the total annual agricultural irrigation consumption from all sources. The terms on RHS represent the water supply to agriculture from GW, DW, and surface-runoff, respectively.

*2*.*2*.*2*.*7 Forestry*. The forestry sector includes all the forests that are managed by the EAD municipality or managed privately in the EAD. They are estimated to account for about 11 per cent of the total water consumption [29, 33]. Forestry water demand depends mainly on the non-potable water sources; namely, GW, and TS. Therefore, the forestry consumption is given by (Eq 12):

$$F_{Consumption\_Total}=GW_{F}+DW_{F}+\;RF\_SR_{F},$$ (12)

where *F*_*Consumption_Total*_ is the total consumption of forestry sector supplied by various sources. The terms on RHS represent the water supply to forestry from GW, DW, and surface runoff, respectively.

#### 2.2.3 Mass balance equations for water transfer subsystem

This subsystem is defined as the transitional storage of water resources. They form the intermediate storage or carrier between the demand and resource subsystems. Here, the study identified three such systems that can be of importance for the mass balance calculations. They are (i) Shallow aquifers (SA), (ii) wastewater treatment system which collects and treats wastewater generated in the EAD, and (iii) the stormwater collection system termed as storm drainage system (SDS) which are designed to collect and discharge the stormwater to the sea.

*2*.*2*.*3*.*1 Shallow aquifer system*. Shallow aquifers form the storage of the GW from which water is abstracted by digging boreholes. The EAD has groundwater aquifers, near Al Ain in the Al Ain Region, and in Liwa in the Western Region. The Liwa Aquifer contains ‘fossilized’ water from 10,000 years ago, the time of the last ice age. The aquifers in the Al Ain Region have benefitted from more frequent recharging due to precipitation in the nearby Hajar Mountains. At the current rate of consumption, the country is utilizing underground water resources more than 20 times as fast as they can be recharged by rainfall [33]. The water used for the irrigation reaches back to the aquifer system as a component, termed as infiltration water, from sectors like agriculture (*A*_*inf-SA*_), forestry (*F*_*inf-SA*_), and amenities (*Am*_*inf-SA*_). Apart from this, the EAD is constructing a man-made aquifer located in Liwa, in the Western Region; that is, a seven-million-gallon underground water storage facility of DW for extreme emergency use [34]. The overall inflow into the aquifer was calculated taking into account all these components and is represented by (Eq 13).

$$SA_{Total-inflow}=A_{inf-SA}+F_{inf-SA}+Am_{inf-SA}+R_{inf-SA}+DW_{inf-SA}+DW_{AR-SA}+RF_{inf-SA}+DA_{inf-SA}+GWE_{inf-SA},$$ (13)

where *SA*_*Total-inflow*_ is the total recharge into the SA, while *A*_*inf-SA*_, *F*_*inf-SA*_, *Am*_*inf-SA*_, R_*inf-SA*_, *DW*_*AR-SA*_, *RF*_*inf-SA*_, *DA*_*inf-SA*_, and *GWE*_*inf-SA*_ represent the infiltration from agriculture, forestry, residential, amenities, municipal, commercial, industrial, leakage of DS water, artificial recharge, natural rainfall recharge, inflow from a deep aquifer, and external aquifer inflow, respectively.

*2*.*2*.*3*.*2 Wastewater treatment plants*. Wastewater treatment plants are the transitional level of converting the wastewater generated to usable treated sewage. The EAD has a well-developed wastewater collection and treatment network, managed by the ADSSC. ADSSC wastewater treatment plants are strategically located in Abu Dhabi City, Al Ain, and few population centers in the Western Region. The current sewerage infrastructure was designed based upon earlier flow projections and is, thus, severely overloaded. Rehabilitation, refurbishment, renewal, and construction of requisite infrastructure are underway to meet the current and future requirements. There are 36 WTP’s operated in the EAD. At all the WTPs wastewater is treated to tertiary level to produce TS, and is primarily used for landscape irrigation. However, only about of 52% of the recycled water is reused for irrigation or other purposes, and the remaining 48% is discharged to the environment [31, 33]. The mass balance of the WTPs were established by (Eq 14), developed by taking into consideration all wastewater components in the EAD.

$$WTP_{Total-inflow}=R_{WTP}+C_{WTP}+M_{WTP}+I_{WTP}+inf_{WTP}$$ (14)

where *WTP*_*Total-inflow*_ is total WTP inflow, while *R*_*WTP*,_ *C*_*WTP*,_ *M*_*WTP*_ and *I*_*WTP*_ are the wastewater generated from all residential, commercial, municipal, and industrial properties, respectively, reaching the WTP. *inf*_*WTP*_ is the infiltration to sewer network systems.

*2*.*2*.*3*.*3 Storm drainage system*. The storm drainage system (SDS) receives and disposes off the stormwater from the rainfalls occurring in the city zones of the EAD, to the sea. Thus, it serves as a transitional system for rainfall occurring in the emirate. The SDS in the emirate is also used for subsurface drainage to control the GW level by removing excess subsurface water, where the maximum level is exceeded. In Abu Dhabi, GW is frequently encountered in construction projects, and needs to be accounted for and dealt with during construction in order to complete the project successfully [35]. This GW removed from the construction sites in the coastal regions of Abu Dhabi because of the high GW level is called “dewatering water”. The SDS has been considered in mass balance calculations, but in the absence of continuous measured value, the data gathered from government entities representatives by organizing workshops, interviews and meeting to discuss the interdependencies, and influence on Abu Dhabi water system, and also from the published document on the SDS of the EAD [36], provided the basis for estimating the SDS data. This is given by (Eq 15).

$$SDS_{Total-inflow}=RF_{SDS}+SA_{inf\_SDS},$$ (15)

where *SA*_*inf−SDS*_ is the infiltration from SA into the SDS system. *SDS*_*Total*−*Inflow*_ represents the total water that is discharged into sea through storm water collection system.

### 2.3 Dynamic water budget model

**Fig 3:**
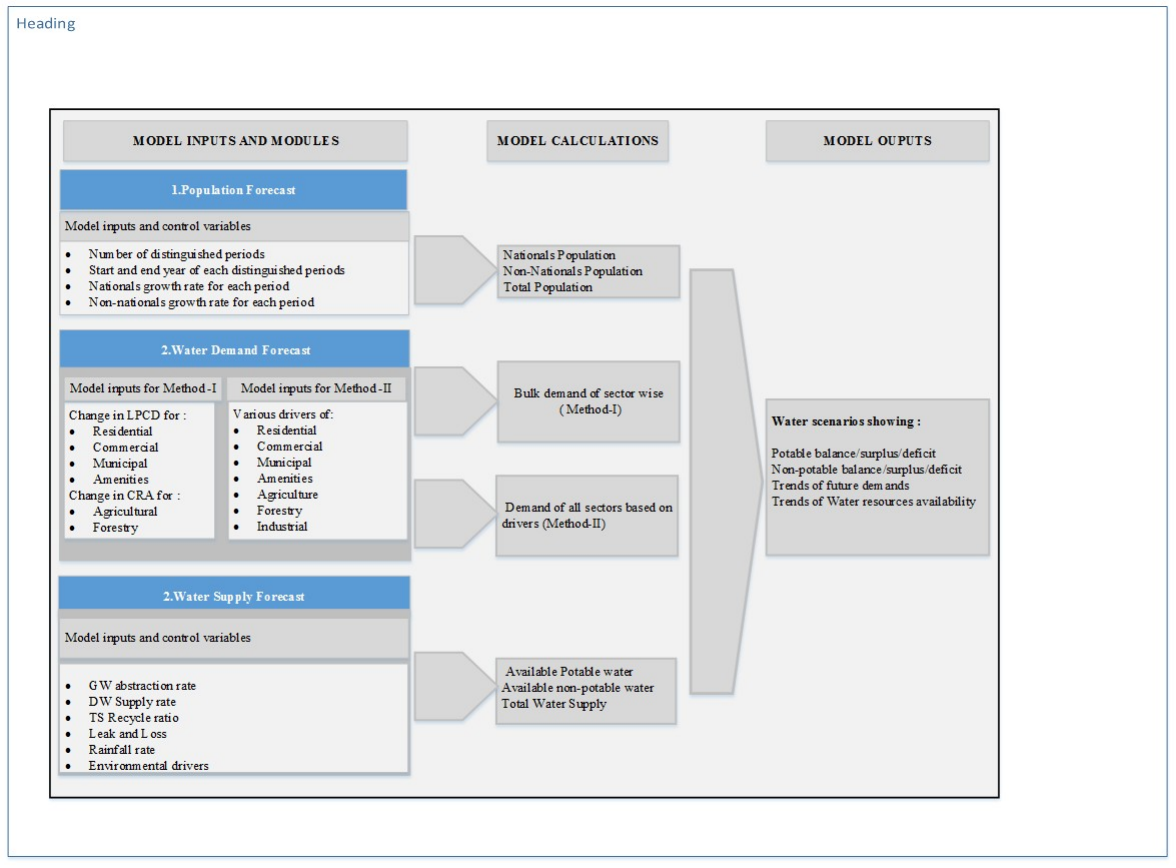
Schematic representation of dynamic model (ADWBM).

The water balance model developed was then used to develop a dynamic model to explore the supply-demand balance over a period of time in the Emirate of Abu Dhabi. The dynamic model, named as Abu Dhabi Dynamic Water Budget Model (ADWBM) is developed as a tool with user-interaction mechanism to generate simulation results for yearly water budgeting. The ADWBM has been designed with three major modules: 1) a population forecast to forecasts yearly population of national and non-nationals separately based on population growth rates, 2) water demand forecast models to forecast sector-wise yearly water demands based on variables, 3) a water supply forecast model to forecast yearly availability of water resources. A set of parameters, variables and operational rules are employed in the dynamic model to run the water balance model for future years. The operational rules are like, to which all demand sectors can a particular water source be supplied, the limit to which a water source can be supplied, the water quality requirements of each demand sectors and so on. These rules are implemented by constructing a number of subroutines in MATLAB to generate yearly water balances until 2050 from the model forecasts of demands and supply. A schematic representation of the ADWBM is shown in Fig 3, while some key parameters used in the model are given in Table 1.

**Table 1:** Sample values and data source of key model parameters.

Model Components	Sample Values	Unit	Sources
GW reserve	220	BCM	[37]
GW extraction rate	2217	MCM/yr	[33]
GW inflow from external aquifers	90–140	MCM/yr	[33]
GW recharge from rainfall	24–30.9	MCM/yr	[29]
Surface-runoff	7.6	MCM/yr	[29]
Leaching rate	5–20	%	[38]
DW Plant Capacities	1280 (2014)	MCM/yr	[32]
DW transmission and distribution loss and leakage	8–10	%	[29]
Evaporation rate	5.3–5.5	mm/day	[29, 39]
Evapotranspiration rate	6.85–8.2	mm/day	[40]
WTP Capacity	408	MCM/yr	[30]
TS use data	284	MCM/yr	[30]
Consumption rates (for all household types)	165–1760	LPCD	[29, 41, 42]
Agricultural consumption data	1716	MCM/yr	[29, 30]
Forestry consumption data	375	MCM/yr	[29, 30, 37]
Amenities Consumption data	112	MCM/yr	[29, 30]
Water requirements for crops	603.7–2040.7	liters/m^2^/yr	[29]
Water requirements for forest	156–221	liters/m^2^/yr	[37]
Amenities water requirement	6.5–10.2	liters/m^2^/day	[43]
Irrigation efficiency	54–90	%	[29, 38, 43]
Offices consumption rate	30.3–56.6	liters/emp./day	[44]
Retail consumption rate	9.9–47	liters/emp./day	[44]
Restaurant consumption rate	9.5–30.8	liters/m^2^/yr	[44]
Hotel consumption rate	130–501	liters/room/day	[44]
Consumption rate in-bay vehicle washing	250–300	liters/vehicle	[44]
Consumption rate by visitors at amenities	3.52–7.04	liters/visitor/day	[43]
Mosque consumption rate	11311–12875	liters/ mosque day	[43]
School consumption rate	15.5–34.1	liters/student/day	[43]
Hospital consumption rate	238–647.6	liters/bed/day	[43]
Government offices consumption rate	0.72–1.34	liters/m^2^/yr	[43]

#### 2.3.1 Population forecast module

In this study, population is considered as the major driving force of water consumption in Abu Dhabi. The study focused on determining various parameters, referred to as “population drivers,” which can predict the future population of Abu Dhabi as accurate as possible. The study found that the Abu Dhabi population follows entirely distinct patterns for nationals and non-nationals. Also, the growth rate will not remain same for the entire planning horizon until 2050 as the Government of Abu Dhabi have set visions for 2020, 2030 and 2050. Each distinguished period follows different growth rates with different values for nationals and non-nationals. Each of them is a population driver and is, therefore, a user input. With these drivers, the model is designed to predict the population of both nationals and non-nationals until the year 2050. A user can develop customized population models for developing future water simulations by considering specific distinguished time periods and growth rates as user inputs.

#### 2.3.2 Water supply forecast module

The future availability of each water resource is dependent on various climatic and environmental parameters, and on the governmental policies based on its visions and sustainability. GW availability is decided by the net annual recharge rate (MCM/yr), net external flow to SA (MCM/yr), and abstraction rates (MCM/yr). Governmental policy sets the allowable abstraction rates per year for a sustainable future. Hence, for groundwater simulation, the model is designed to accommodate three parameters: net annual recharge rate, net external flow to the SA, and abstraction rates to predict the future GW situation. Based on the latest estimated values available, the baseline values used in the model are 200 BCM for the current GW reserve, 110 MCM/yr for net recharge rate, 0 MCM/yr for net external flow to SA, and 2,200 MCM/yr for current GW abstraction rate.

The parameters of the future desalination are subject to governmental policies. In the model, “annual desalination capacity” and “leakage and loss” are the two key parameters related to DW. Currently, 10% is considered as the baseline value for “leakage and loss” based on [33].

TS availability is dependent on potable water consumption and the wastewater generated. Thus, to forecast the future TS generation, the parameters included in the model are potable water return ratio (*PWR*), infiltration rate to sewer line, and recycle ratio of produced TS (*RR*_*TS*_). PWR is defined as the ratio of wastewater reaching at the inlets of WTP to the total potable water consumption. *RR*_*TS*_ is dependent on the quality of the water produced and the capacity of the TS distribution system. The values of parameters are included in the model based on the data obtained from relevant entities and operators of wastewater treatment plants and pumping facilities in the EAD.

For rainfall, surface-runoff, infiltration rate, and evaporation rate are the key parameters. All relevant data for forecasting were collected from various sources like historical records, technical reports, official and government publications.

#### 2.3.3 Water demand forecast module

The ADWBM is constructed with two methods to forecast water demands. Method-I is a simple approach by which bulk demand of a sector can be forecasted using a single-coefficient. In Method-II, the determinants contributing to or driving the consumption in a sector, here referred to as demand drivers, form the basis for demand forecast.

*2*.*3*.*3*.*1 Method-I for water demand forecast*. Water demand functions were constructed which relate bulk demand of sector to a unit consumption rate. The per-capita–demand (*PCD*) expressed in liters per capita per day (*lpcd*) is the coefficient used for modeling water demands by population dependent sectors. Strong relationship between the population, and the residential and commercial demands is obvious; it still exists with municipal and amenities demands and was statistically determined. These demands showed positive linear relationship with population and *R*^*2*^ value was found to be between 0.78 and 0.96, with residential and amenities, having highest and lowest values, respectively. Water demand is calculated as a product of the overall population “*P*” and the “*PCD*” for the respective sectors, as represented by (Eq 16). The *PCD* for each population dependent demand sectors were determined from the historical data of water consumption by different sectors in the EAD.

$$D_{BPD}=\;P*PCD_{PD}*365*10^{-9},$$ (16)

where, *D*_*BPD*_ is the annual bulk demand of population dependent sectors expressed in MCM/yr, *P* is the forecasted population, and *PCD*_*PD*_ is the *lpcd* determined for respective population related sectors.

Agricultural and forestry demands are dependent on the area under irrigation. The unit consumption rates per area by these sectors formed the basis of demand estimation by this method. Historical data of yearly consumption by these sectors and area under irrigation for respective sectors were statistically analyzed to the estimate the “consumption rate per unit area” (*CPA*) for the agricultural and forestry sectors. The demands by these two sectors are given by (Eq 17):

$$D_{BNPD}=\;A_{Di}*\;CPA_{Di}*365*10^{-9},$$ (17)

Where *D*_*BNPD*_ is the annual bulk demand of sectors (A and F) in MCM/yr, *A*_*Di*_ is the irrigated area in *m*^*2*^ under a respective sectors and *CPA*_*Di*_ is consumption rate of respective sector in liters/m^2^/day.

Therefore, Method-I in ADWBM is designed to forecast demand based on *PCD* for population dependent sectors and *CPA* for population independent irrigation demands.

*2*.*3*.*3*.*2 Method-II for water demand forecast*. In Method-II, components contributing to or driving the consumption in the sector, here referred to as demand drivers, are the core of demand forecast. Several drivers exist for each demand sector, and their details are needed to enable a better estimation (projection) of each type of demand as per its categorized drivers. This method of modeling is preferred due to its comprehensiveness and level of aggregation, as this yields more representative figures of sub-sectors within a sector and, eventually, better results for future planning. The equations to structure water demand forecasting based on drivers is drawn from the experience of forecasting for the majority of United Kingdom water companies over the last three decades but has been modified to reflect Abu Dhabi conditions and are derived by making necessary adjustments to the (Eq 16). This involved identification of demand drivers of each sector in Abu Dhabi and developing demand equations based on them.

*2*.*3*.*3*.*3 Residential*. The residential consumption in EAD was aggregated into five components (drivers) associated with three types of housing: shabiyat, villas, and flats. Shabiyat are one-story low-income family houses inhabited by an unaccounted number of residents. The equations developed to structure residential water demands in the EAD based on drivers are explained through Eqs (18)–(23).

Since Abu Dhabi has a population structure with two very clear population groups, nationals (n), and non-nationals (nn), the PCD based demand equation; (Eq 16) was adjusted to reflect this change in the residential demand as in (Eq 18). This helps to estimate the residential demand for nationals’ and non-nationals’, separately.

$$D_{R}=\;(P_{n}*PCD_{R-n}+\;P_{nn}*PCD_{R-nn})*365*10^{-9},$$ (18)

where *P*_*n*_ and *P*_*nn*_ are nationals’ and non-nationals’ population, respectively. *PCD*_*R-n*_ and *PCD*_*R-nn*_ are their respective consumption rates in *lpcd*.

However, the consumption rates used in (Eq 18) are determined either from aggregate data (population and supply to residences) or by sampling or surveys of household numbers if metered data is to be used. The basic data on direct consumption is metered data, which is collected at the household level. Such data can be used to determine the total demand from the aggregation of consumption by household types in the EAD, where *N*_*j*_ is the number of households of type *j*. Thus (Eq 19) is obtained as follows:

$$D_{R}=\sum_{0}^{j}N_{j}CR_{j}*365*10^{-9},$$ (19)

in which *CR*_*j*_ is the consumption rate by *j* type households.

Furthermore, considering nationals and non-nationals as separate modeling elements in different household types, it was further modified as (Eq 20).

$$D_{R}=(\sum_{0}^{j}(N_{jn}CR_{jn})+\;\sum_{0}^{j}(N_{jnn}CR_{jnn}))*365*10^{-9}{}_{,}$$ (20)

where *N*_*jn*_ and *N*_*jnn*_ are the number of *j* type household occupied by nationals and non-nationals, respectively. *CR*_*jn*_ and *CR*_*jnn*_ are consumption rate by *j* type households occupied by nationals and non-nationals, respectively.

Finally, recognizing the need to differentiate the indoor consumption, “*i*”, and outdoor consumption,”*x*”, at each household type yielded (Eq 21), which is the residential demand equation that comprises all the drivers of residential sector.

$$D_{R}=(\sum_{0}^{j}N_{jn}(CR_{jni}+CR_{jnx})+\sum_{0}^{j}N_{jnn}(CR_{jnni}+CR_{jnnx}))*365*10^{-9},$$ (21)

*2*.*3*.*3*.*4 Municipal*. The key drivers of municipal demands in the EAD are identified to be the government offices, hospitals, schools, mosques, and visitors to recreational facilities. The unit consumption rates for these drivers per head or per area, whichever is applicable formed the basis for the estimation of municipal demand using Method-II. The municipal demand equation developed on these drivers is given in (Eq 22).

$$D_{M}=(Ar_{gov-off}*\;CR_{M-gov-emp}+\;N_{mq}*CR_{M-mq}+\;N_{hs-bed}*CR_{M-hs}{}_{}+CR_{M-sc}*N_{st}+\;N_{vs}*\;C_{M-vs})*365*10^{-9},$$ (22)

where N_*hs-bed*_, *N*_*mq*_, *N*_*st*_ and *N*_*vs*_ are total number of hospital beds, mosques, students, and visitors to recreational facilities. *Ar*_*gov-off*_ is the gross floor area of governmental offices in *m*^*2*^. The other parameters (consumption rates) in the equation are: *CR*_*M-gov-off*_*—*liters/m^2^ /day, *CR*_*M-mq*_*—*liters/m^2^/day, *CR*_*M-hs*_*—*liters/hospital bed/day, *CR*_*M-sc*_—liters/m^2^/day, and *CR*_*M-vs*_—liters/visitor/day.

*2*.*3*.*3*.*5 Commercial*. Commercial sector in the EAD mainly included hotels, restaurants, cafeterias, car washes, and laundries. The demand equation was therefore developed as follows (Eq 23):

$$D_{C}=(N_{off-emp}*\;CR_{C-off}+N_{ret-emp}*CR_{C-ret}+\;Ar_{res}*CR_{C-res}{}_{}+N_{hr}*O_{hr}*\;CR_{C-hr}+\;N_{cw}*\;CR_{C-cw})*365*10^{-9},$$ (23)

where, *D*_*C*_−total annual commercial demand in MCM/yr, *N*_*off-emp*_−number of office employees, *CR*_*off-emp*_−consumption rate per office employee in l/employee/day, *N*_*ret-emp*_−number of retails employees, *CR*_*C-ret*_*—*consumption rate per retail employee in l/employee/day, *Ar*_*res*_*—*gross area of restaurants in m^2^, *CR*_*c-res*_−consumption rate per floor area in l/m^2^/day, *N*_*hr*_−total number of hotel rooms available for occupancy, *CR*_*C-hr*_*—*consumption rate per hotel room occupied l/occupied room/day, *O*_*hr*_—occupancy rate of hotel rooms, *N*_*cw*_—total number of all vehicle washes in all car wash units, and *CR*_*C-cw*_*—*consumption rate per vehicle wash in liters/vehicle.

*2*.*3*.*3*.*6 Industrial*. As the growth of industries in EAD are driven by the governmental policies and visions, with no drivers information, the forecast equation in the model has been designed to calculate the future demands based on the change in rate of annual industrial consumption, taking 20 MCM/yr as the base value. The change in rate; increase or decrease, is dependent on the governmental economic strategies.

*2*.*3*.*3*.*7 Amenities*. Amenities demand is governed by key drivers, namely, amenities areas and irrigation efficiency for two types of lands: parks and ornamental fields. From this, (Eq 24) was developed to forecast the amenities water demand using Method-II.

$$D_{Am}=(\sum (AmR_{k}*Ar_{k})/IE_{Am}+L_{r})*10^{-6},$$ (24)

in which *D*_*Am*_ is the annual water demand of amenities in MCM/yr, *k*- type of amenities, *AmR*_*k*_ *is y*early amenities water requirement per unit area for type *k* amenities, *Ar*_*k*_ is the irrigated area of *k* type amenities, *IE*_*Am*_ is the irrigation efficiency for landscape irrigation and *L*_*r*_ is the leaching requirement.

*2*.*3*.*3*.*8 Agricultural*. In the model, the water demand by the agricultural sector is forecasted by Method-II, in based on the drivers identified as cultivated area of each type of the crop, the irrigation requirement of each crop type, the irrigation efficiency and leaching requirement of the agricultural lands. Therefore, the agricultural demand equation was developed as (Eq 25) given below:

$$D_{A}=\;(\sum (CWR_{i}*\;Ar_{i})/IE_{A}+L_{r})*10^{-6},$$ (25)

in which *D*_*A*_ is total annual water demand of agriculture, *CWR*_*i*_ is the yearly crop water requirement for type *i* crop per unit area, *Ar*_*i*_ is the area under cultivation for *i* type crop, *IE*_*A*_ is the irrigation efficiency, and *L*_*r*_ is the leaching requirement.

*2*.*3*.*3*.*9 Forestry*. The irrigational demand of forestry sector is governed by the occupied area, consumption rates in the eastern and western regions, and irrigation efficiency in forest lands of the EAD. Therefore, the forestry demand equation was developed as in (Eq 26):

$$D_{F}=(\sum (FWR_{r}*Ar_{r})/IE_{F}+L_{r})*10^{-6},$$ (26)

in which *D*_*F*_ is the total annual water demand of forest, *FWR*_*r*_ *is y*early forestry requirement per unit area for region *r*, *r* refers two regions where forests are located, *Ar*_*r*_ is the irrigated forest area in region *r*, *IE*_*F*_ is the irrigation efficiency and *L*_*r*_ is the leaching requirement.

#### 2.3.4 Future simulations development and evaluation using ADWBM

The implementation of the ADWBM is that (i) the user can develop any number of computational simulations by varying the control parameters (drivers such as policy inputs and water conservation strategy consumption parameters) used for the forecast, (ii) it hosts a module to do prioritized allocations of available potable water (DW) to potable demand sectors (residential, commercial, municipal, and industrial) and the non-potable water (TS and GW) to non-potable demand sectors (agriculture, forestry, and amenities), and (iii) determines the required percentage reduction in water consumption by each demand drivers, which details water reductions needed in each demand sector to achieve a BWB till 2050. The BWB refers to conditions where water resources are available to meet expected demands with no encountered shortage in any sector. In each module, a user can change the input values to investigate the impacts on future water management scenarios.

As part of this study, two workshops were organized with governmental entities and stakeholders to discuss and gather more accurate data relevant to the model development. The ADWBM is equipped to update with any latest data whichever becomes available in future, which makes it an adaptive tool.

## 3. Results and discussion

### 3.1 Model calibration and validation

Model calibration and validation was done to ensure that the forecasting methodology adopted is acceptable [45]. In this study, calibration is carried out by which various model parameters were adjusted and optimized in order to improve the simulation results. Various model outputs include forecasted demands of the sectors-residential, commercial, municipal, agricultural, forestry, amenities, and the wastewater generation (TS availability). The calibration mainly focused on adjusting and optimizing the values of the parameters (drivers) of these demand sectors and TS. The parameters were adjusted until a reasonable statistical agreement between the observed and simulated values was obtained. For instance, the residential demand was calibrated by using following parameters: indoor consumption rate in shabiyat, outdoor consumption rate in shabiyat; indoor consumption rate in villas, outdoor consumption rate in villas, and consumption rate in flats. Similarly, for non-potable sectors, calibration was carried out by adjusting their respective drivers. For the TS, as the model is forecasting the total wastewater that will be treated at WTPs to produce TS, the calibration was based on adjusting two parameters; the potable water return ratio, and the infiltration rate into the sewer system. The optimized and calibrated values of parameters are summarized in the Table 2. This could serve as baseline values for developing water scenarios, as discussed in [46] to set up the water vision for Abu Dhabi.

**Table 2:** Optimized values of parameters after calibration.

Demand sector	Drivers	Value (unit)
Residential	Shabiyat Indoor	320 lpcd
	Shabiyat Outdoor	1280 lpcd
	Villas Indoor	240 lpcd
	Villas Outdoor	960 lpcd
	Flats	400 lpcd
Commercial	Office Employees	56 liters/emp./day
	Retail Employees	47 liters/emp./day
	Restaurants	30 l/m^2^/day
	Hotel Rooms	330 liters/room/day
	Carwash	284 liters/vehicle
Municipal	Government offices	2.2 liters/m^2^/day
	Mosques	12,774 liters/mosque/day
	Schools	34 liters/student/day
	Hospitals	259 liters/bed/day
Agricultural	Water requirement for fruit crop	2040.7 liters/m^2^/yr
	Water requirement for field crop	603.7 liters/m^2^/yr
	Water requirement for vegetable crop	605.6 liters/m^2^/yr
	Irrigation efficiency for agriculture field (%)	54
Forestry	Water requirement for forest-Western Region	156 liters/m^2^/yr
	Water requirement for forest-Al Ain Region	221 liters/m^2^/yr
	Irrigation efficiency for forest land (%)	56
Amenities	Water requirement for amenities irrigation	9.6 liters/m^2^/day
	Irrigation efficiency for amenities (%)	54
TS	Potable water return ratio (PWR)	0.286
	Infiltration rate to sewer line	10%

The period from 2005 through 2014 was used for calibration while the period 2015–2019 was chosen for validation. Three statistical parameters namely, mean of relative error (MRE), Nash-Sutcliffe Efficiency (NSE) and coefficient of determination (R^2^) were used to assess the performance. MRE gave the relative differences between the model and actual values. NSE was used to determine accuracy of the model. NSE takes value in the range -∞ to 1, with closer the value to 1, the more accurate the simulated value. R^2^, a number between 0 and 1, describes the collinearity between the model and actual values. Closer the value to 1, the model simulates the system well.

**Fig 4:**
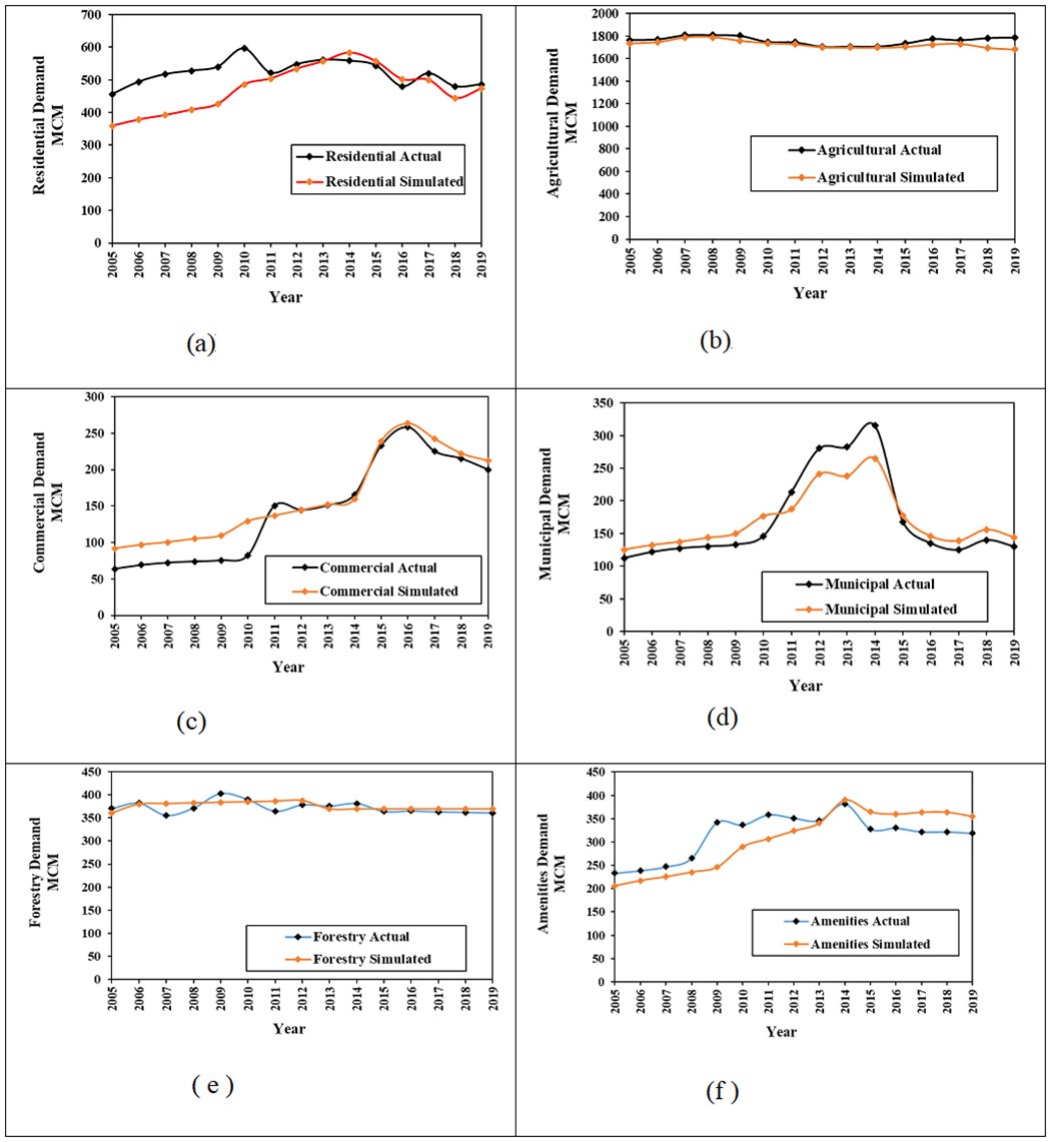
Comparison of simulated results and historical (actual) data. **(A)** Residential. **(B)** Agricultural. **(C)** Commercial. **(D)** Municipal. **(E)** Forestry **(F)** Amenities.

The analysis of performance using the aforementioned statistical metrics are presented in Table 3. It shows that MRE ranges from -12.3 to 15.17%, R^2^ is between 0.674–0.973, and NSE is between 0.619–0.933. The plots (Fig 4) show that the model was able to reproduce the results that fit well with the historical values. Municipal, amenities and commercial sector showed a relatively low value for the NSE; 0.619, 0.623 and 0.645, respectively. These comparatively low values are due to inaccuracies in drivers’ data for these sectors, which when updated could improve the model prediction. However, a value of above 0.5 for NSE is considered as satisfactory [47]. Therefore, the overall results of the calibration and validation showed that the model is able to reproduce the water demand and supply trends adequately well and is suitable for use.

**Table 3:** Statistical analysis of calibration performance.

Model Parameters	Statistical Analysis Values		
	MRE %	R^2^	NSE
Agricultural Demand	-1.14	0.842	0.794
Residential Demand	-11.52	0.902	0.875
Municipal Demand	-12.3	0.718	0.619
Commercial Demand	15.17	0.876	0.645
Forestry Demand	0.966	0.973	0.933
Amenities Demand	-7.58	0.674	0.623
TS Availability	6.08	0.879	0.780

### 3.2 Future simulations

Results of two simulations depicting the future water situation in Abu Dhabi are discussed here.

#### 3.2.1 Baseline (BL) simulation

**Fig 5:**
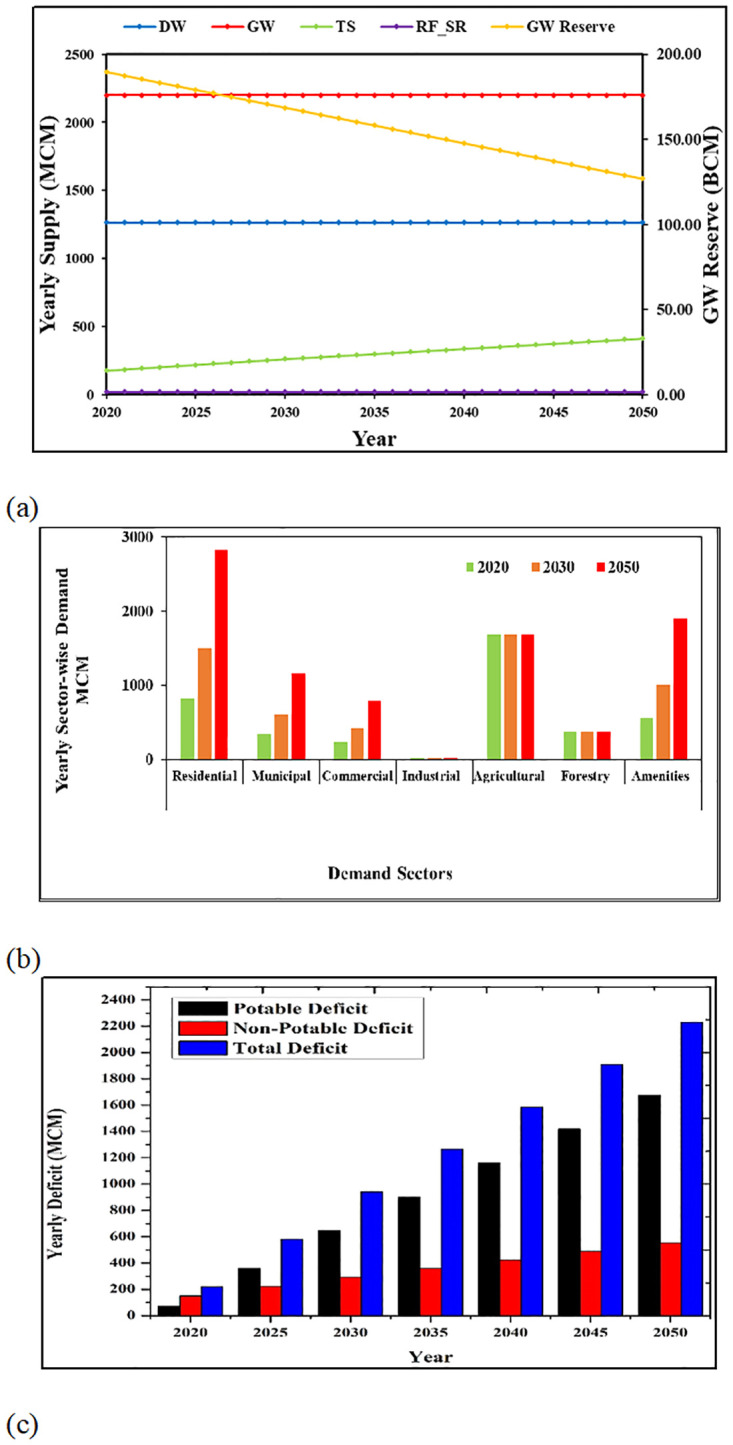
Results of BL simulation. (A) Supply from different supply sources and trend of GW reserve over years. (B) Water demand in all sectors under the BL simulation for 2020 (first bars), 2030 (second bars), and 2050 (third bars). (C) Increasing trend of water deficit over years for BL simulation.

The BL simulation is a reference simulation, which is developed to represent the continuation of current water demand and supply trends in the EAD. Under this simulation, the consumption rates are assumed to remain unchanged as in the baseline year 2015 but the population will continue to grow. In this simulation, Abu Dhabi population is assumed to grow in line with a balanced environment and a gradual economic development. Therefore, under BL simulation, population growth pattern in Abu Dhabi will follow three distinct periods of growth between 2015–2050: the periods of 2015–2020, 2021–2030, and 2031–2050. The annual growth rates for these distinguished periods represent the growth rates of “*Scenario F*’ in [48] and “*SSP5 Scenario*” in [49]. This represents a condition of conventional population growth in Abu Dhabi. This simulation is developed to illustrate a situation in the EAD with no improvement in water supply infrastructures with respect to the baseline year. Furthermore, the BL simulation assumed that no restriction will be imposed on groundwater extraction from the baseline value. Also, the change in climate is unlikely to make a severe change on water resources by 2050 [29, 50]. Therefore, in this simulation the climatic factors like rainfall and temperature are kept unchanged from the current pattern for the planning horizon. Under BL simulation, water demand will grow with time for population dependent sectors while the water demands by other sectors will remain same as the baseline consumption throughout the planning horizon. The consumption rates per sector used for BL simulation are baseline values. The yearly supply from various sources considered in the BL simulation is shown in Fig 5A.

The BL simulation in ADWBM revealed that a balanced water budget could not be achieved under this simulation. The annual sector-wise demand is shown in Fig 5B and it can be seen that overall annual demand exceeds 6000 MCM by 2050. This increase will cause the potable and non-potable demand sectors to face a deficit in coming years. For BL, the model predicts a shortage in potable and non-potable water supply as early as 2020, and continue to increase thereafter until 2050. This trend of water deficit over years is shown in Fig 5C. The GW reserve is overexploited under BL simulation and will result in an alarming decline by the year 2050 (Fig 5A). As the water deficit is increasing rapidly, the EAD must make major changes in governmental policies and regulations in order to prevent water crisis in the future if the current trend of water resources management is continued.

The BL simulation needs modifications in the consumption patterns by different sectors to have a balanced water budget for the same population growth and for the time horizon until 2050. Therefore, the BL simulation is re-run with changes to check how a BWB can be achieved by controlling the water supply, and it is considered as a new simulation.

#### 3.2.2 Controlled Supply (CS) simulation

This simulation demonstrates how the water deficits predicted in BL simulation can be avoided. The main objective of this simulation is to determine an optimal solution for achieving water security in the EAD, with the same population and economic growth as in BL simulation. Therefore, this simulation is a target-based simulation. Iterative simulations were carried out to find out the corresponding conservations to be implemented in each demand sectors to avoid any annual water deficit until the year 2050 if the supply from available sources is controlled. Under this simulation, assumptions on water supply sources were made on a conservation and sustainable approach by targeting GW abstraction less than or equal to the natural GW recharge rate of 110Mm^3^/yr by 2050, limiting desalination capacity expansion to the minimum, and maximization of produced TS use by 2050. Moreover, it is assumed that Abu Dhabi will have an increased rainfall by 20%, by its sustainable policy which targets an increase in rainfall through artificial rain program using harmless natural salts [51]. The climate change strategy that was incorporated into Abu Dhabi Environment Vision 2030 [46] is also taken into account in this simulation. Iterative simulations were then conducted to find the optimized reductions needed for different sectors to achieve the BWB. CS achieved a BWB throughout the entire period (no shortage) by applying reductions to the baseline consumption rates.

**Fig 6:**
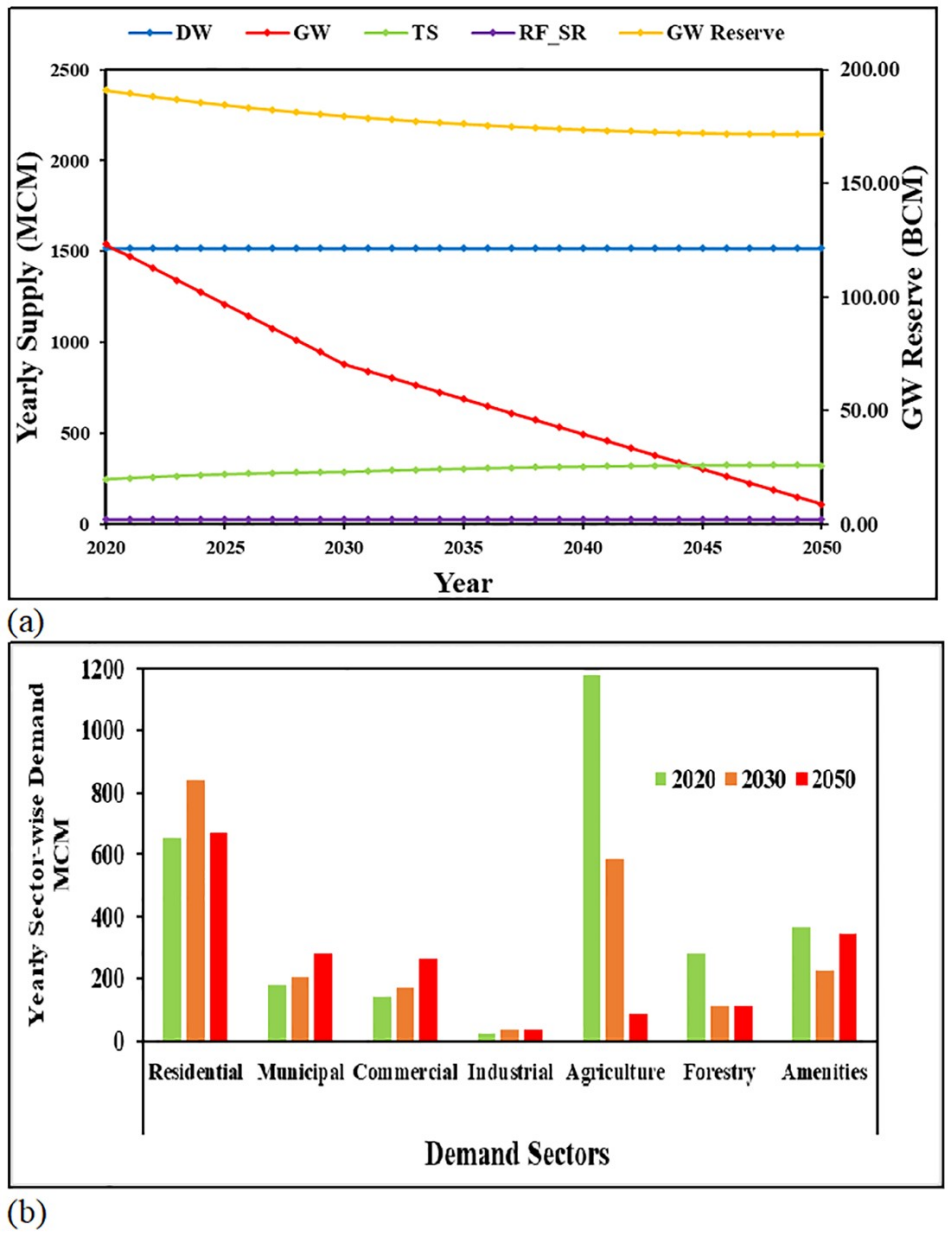
Results of CS simulation. (A) Supply from different supply sources and trend of GW reserve over years. (B) Water demand in all sectors under the CS for 2020 (first bars), 2030 (second bars), and 2050 (third bars).

Simulation results showed that large percentage reductions are needed to achieve a BWB. The required reduction is expressed as a percentage of the baseline consumption rates given in Table 4. The reductions for population-related demands (residential, commercial, municipal, and amenities) refer to consumption rates, lpcd, while the reductions for agriculture and forestry refer to consumption rates in year. The trend of supply by all sources and dynamics of water demand by all sectors until 2050 is illustrated in Fig 6A and 6B, respectively. For CS, the strategic groundwater reserve remains conserved with marginal annual GW abstraction (Fig 6A).

**Table 4:** Reductions needed in demand sectors for CS for achieving a BWB.

Demand Sector	Baseline values	Target Reduction Values (% reduction from baseline)		
		2020	2030	2050
Residential	610 lpcd	549 (10)*	427 (30)	256 (58)
Commercial	170 lpcd	119 (30)	93 (45)	93 (45)
Municipal	250 lpcd	150 (40)	112 (55)	110 (56)
Agriculture	1680 MCM/yr	1176 (30)	588 (65)	84 (95)
Forestry	375 MCM/yr	281 (25)	112 (70)	112 (70)
Amenities	410 lpcd	307 (25)	123 (70)	123 (70)

*Values in parenthesis are in reduction percentages required with reference to baseline values.

**Fig 7:**
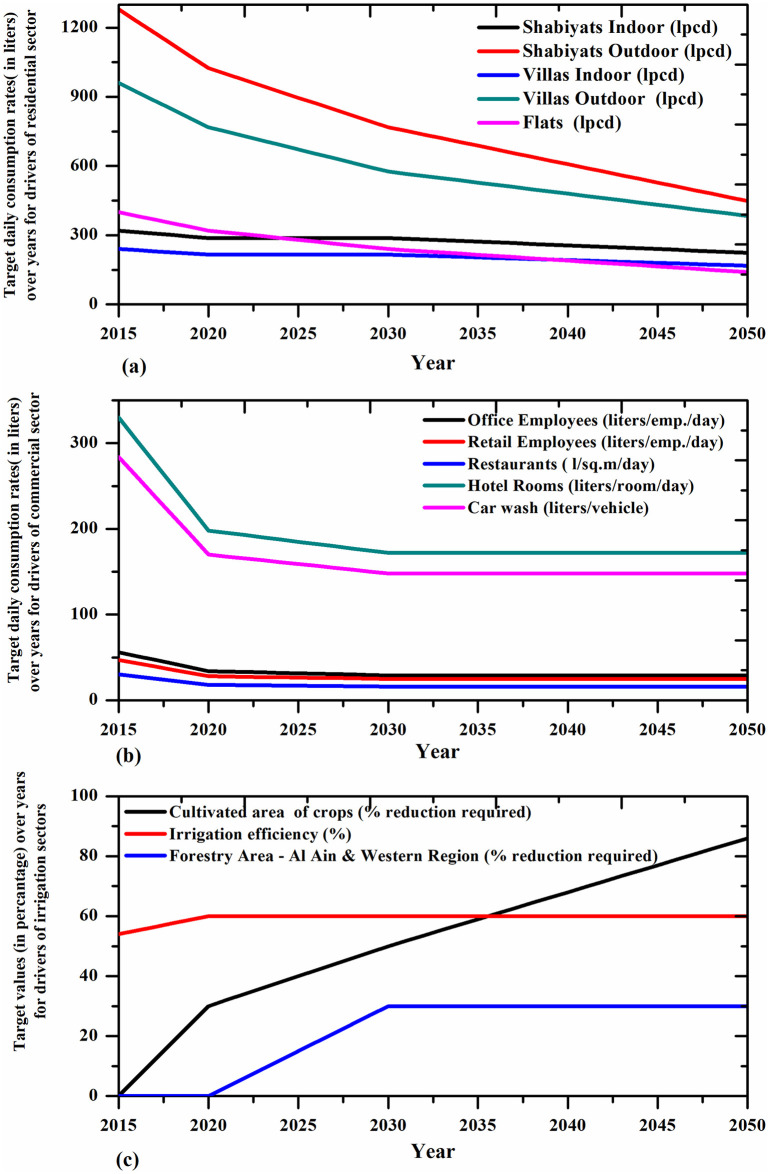
Required reductions in consumption at drivers’ level for major demand sectors in the CS simulation. (A) Target reduction in residential sector. (B) Target reduction in commercial sector. (C) Target reduction in irrigation sectors (Agricultural and Forestry).

Further, in order to demonstrate how much of conservation or reduction in demand drivers or sub-sectors are required for controlling the majority of water consumption in a particular demand sector, a second level of simulations were carried out. That is, in this simulation step, the driver-based methodology was followed, and the simulation presented the results as target values to be achieved for drivers from baseline values to achieve a BWB. The simulation results of drivers of four major demand sectors are presented in Fig 7.

For the residential sector it can be seen that extreme reductions are required in outdoor consumption, by the year 2050 Fig 7A. The target unit consumption rates to be achieved for the commercial consumption drivers are shown in Fig 7B. Compared to residential sector, commercial sector requires only slighter reductions in drivers’ consumption rates.

It was found that it is not feasible to achieve BWB without very large reductions in the irrigation demands of agricultural and forestry sectors. Therefore, to reduce the consumptions in these sectors the options available are to increase the irrigation efficiency without compromising on the plant water requirements, and to reduce the area under these sectors. Simulation results showed that improving the irrigation efficiencies in agriculture and forestry sectors alone will be enough to achieve a BWB until the year 2020. However, to achieve a BWB until 2050, the simulation results showed that reduction in area under these sectors are required. For agriculture sector the required reduction in area from the baseline value is 86% while this figure is 60% for forestry sector Fig 7C.

Controlled supply simulation is recommended to be adopted because of reasonable as well as achievable percentage reductions needed to apply for different demands at different years (Fig 7). Thus, from the scenario results, only through strict conservation strategies can water demand-supply balance be achieved in Abu Dhabi. The reductions in water demand require involvements from the government entities to implement specific programs targeting water conservation. This could be in the form of preparing long term programs for educating the citizens on the importance of water conservation, technical and legislative interventions to reduce water demand, and so on. Thus, CS can lead to the realization of sustainable Abu Dhabi through conservation approach.

### 3.3 Sensitivity analysis

**Fig 8:**
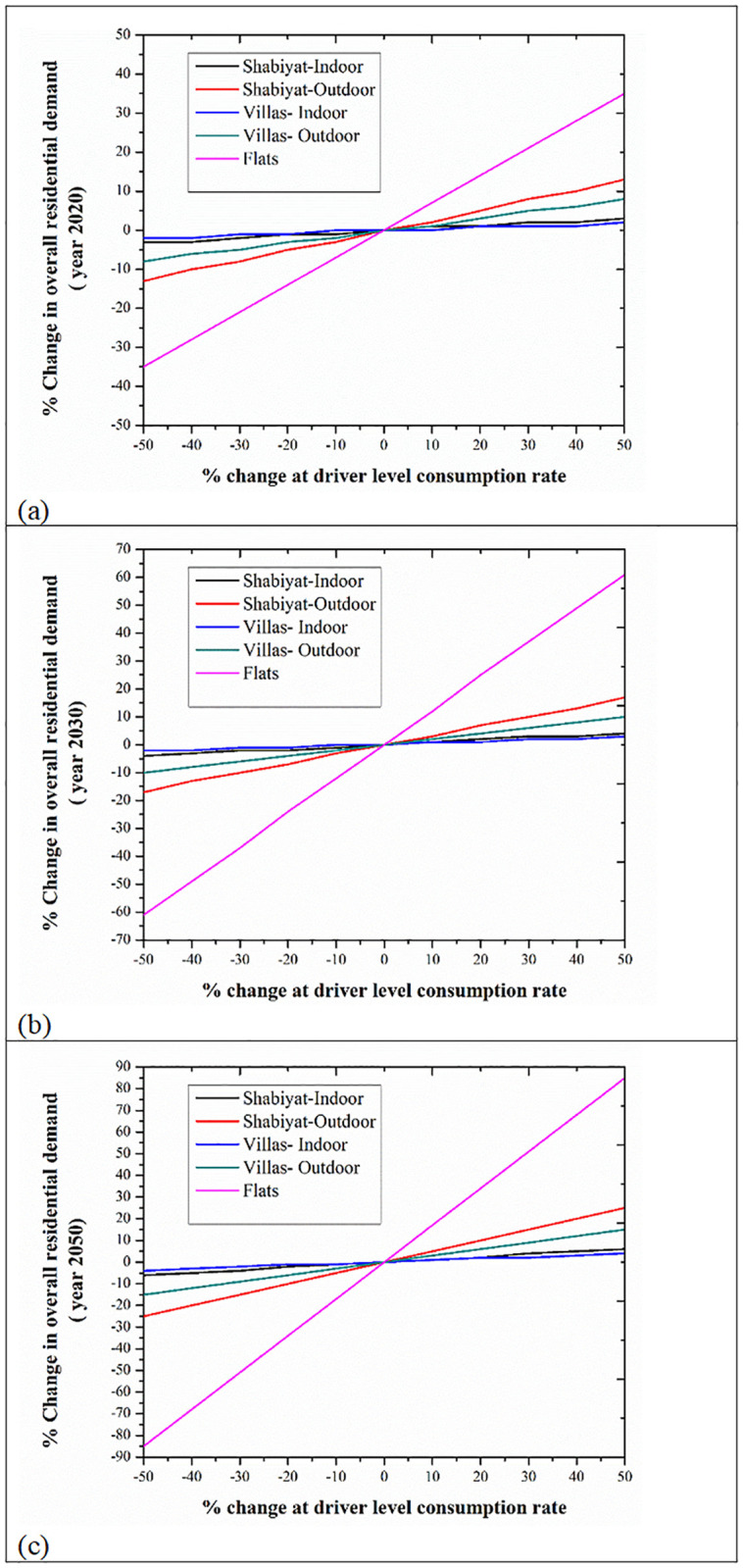
Sensitivity analysis-effect of drivers on residential demand. **(A)** For year 2020. **(B)** For year 2030. **(C)** For year 2050.

Since there are many drivers associated with different demand sectors, it is necessary to identify the drivers that have the largest influence on the calculated demand so that future efforts can be focused on gathering data for those drivers. Therefore, a sensitivity analysis was conducted to evaluate the impact of drivers on the calculated consumption. This analysis was performed separately for each demand sector by changing the value of an individual driver (% increase and decrease), keeping other drivers unchanged, and reporting the percentage change of that demand sector at years 2020, 2030, and 2050. It is worth mentioning that it was assumed that changing the driver(s) of any demand sector does not affect other demand sectors. The residential sector is used as an example to explain the sensitivity analysis approach. It shows that the input parameters that affect the residential demand mostly in all three-time horizons (2020, 2030, and 2050), are the flats water consumption followed by the Shabiyat’ outdoor water consumption. The villas outdoor consumption acts as the third most influential parameter for all the three time horizons considered. The effect of each driver (while other drivers remain the same) on the residential demand for 2020, 2030, and 2050 is shown in Fig 8. It is identified that though the flats water consumption rate is relatively low, the high population in this category of dwelling makes it the most influential input driver. The changes in demand increase with time; that is, changes in 2050 are larger than those in 2030 and 2020.

A similar approach was followed for all other sectors having detailed drivers’ data. In the municipal sector, the government offices area and its consumption rate are the drivers that mainly influence the municipal water demand in 2020, 2030 and 2050. According to the effect on the commercial water demand, the most influential input parameters for all the three time horizons are the water consumptions by restaurants. The retail employee and the office employee number have the similar impact on the demand. The water consumption for car wash and in hotel have minimum effect on the commercial water demand. In the agriculture sector, the controllable driver, namely, irrigation efficiency and area under cultivation (fruits, field and vegetables) affect the agriculture demands significantly. There are two controllable drivers in the forestry sector. These are the total area of forestry (region-wise) and the irrigation efficiency. Both drivers significantly affect the overall forestry water consumption in the 2020, 2030 and 2050. In the amenities sector, the irrigated area is broadly divided into two categories, park and ornamental areas. Therefore, the input drivers, namely, amenities area (park area and ornamental area), consumption rates and their irrigation efficiencies. It was observed that the amenities area and irrigation efficiency affect the overall amenity water demand without altering the consumption rate.

## 4. Conclusions

This study developed a numerical tool to produce as accurate figures as possible for water supply-and-demand in the EAD until 2050 for planning and accommodating actions needed to eliminate the potential shortage. Two future water simulations (until 2050) were then constructed, emphasizing conservation options, to represent different future water conditions. Of the two simulations discussed in this study, BL showed high levels of vulnerability with water shortage to soon occur for both potable and non-potable water demand. Hence, achieving a balanced water budget was illustrated through a simulation named ‘Controlled Supply’, which demonstrated that water balance is achievable only by implementing strict measures in consumption patterns in addition to the controlled supply of water. The required target reductions in water consumption rates in different demand sectors were simulated which was further analyzed to obtain the required reduction percentages by individual demand drivers so that the policymakers can determine which sub-sectors (demand drivers) need to be prioritized for consumption reductions. Demographic conditions, governmental strategies and visions, and environmental considerations were the central to the analyses.

The study revealed that the majority of the population has a water-intensive lifestyle. This finding is reflective of residential usage as shabiyat and villa residents have high outdoor consumption. Thus, the EAD contrasts with other regions’ trends, where consumption has stabilized or declined [52]. Even if the expected declines in population growth rates prevail from 2015 to 2050 for both nationals and non-nationals, it is unlikely that this will be enough to curtail demand as consumption increases rapidly under the BL simulation. The Emirate must make major changes to pursue the consumption pathways modeled by possible BWB simulations (like controlled supply), different from the BL simulation. Initiatives such as ‘Environment Vision 2030 [48]’ suggest a step toward a sustainable vision. However, efforts need to be maximized at all levels to realize this vision. Frequent updates to the Water Resource Management Strategy [33] may also contribute to these efforts, although effective communication of this strategy may be as important as the strategy itself. Furthermore, long-term thinking beyond 2030, by various stakeholders, is critical to ensure that water consumption rates are met and maintained at a sustainable level, ensuring future populations can meet their needs at reasonable costs. Societal adaptations will prove to be vital as previously discussed, which includes a review of outdoor water usage, the addition of efficient household appliances, and the removal of government subsidies. Despite supply augmentation being considered, wastewater reuse and desalination expansion should not be prioritized. Targeting consumption, which is the source of the problem, should be given precedence. The simulations developed could be advanced further with new knowledge and data availability over time.

## Abbreviations

A_inf-SA_: Infiltration to SA from agriculture
AmR_k_: Water requirement for k type amenities
Am_inf-SA_: Infiltration to SA from amenities
Ar_gov-off_: Gross floor area of governmental offices
Ar_i_: Area of *i* type irrigated vegetation
Ar_k_: Area of *k* amenities facilities
Ar_r_: Area of forest in *r* region
Ar_res_: Average area of restaurants
BCM: Billion cubic meter
BCM/yr: Billion cubic meter per year
C_M-vs_: Consumption rate per visitor
CPA_Di_: consumption rate per area for irrigation demand sectors
CR_C-cw_: Consumption rate per vehicle
CR_C-hr_: Consumption rate per hotel room occupied
CR_C-off_: Consumption rate per office employee
CR_C-res_: Consumption rate per restaurant area
CR_C-ret_: Consumption rate per retail employee
CRj: Consumption rate by *j* type households
CRjn: Consumption rate by *j* type household by nationals
CRjni: Indoor consumption rate by *j* type household by nationals
CRjnn: Consumption rate by *j* type household by non-nationals
CRjnni: Indoor consumption rate by *j* type household by non-nationals
CRjnnx: Outdoor consumption rate by *j* type household by non-nationals
CRjnx: Outdoor consumption rate by *j* type household by nationals
CR_M-gov-emp_: Consumption rate per government office employees
CR_M-hs_: Consumption rate per hospital bed
CR_M-mq_: Consumption rate per mosque
CR_M-sc_: Consumption rate per school students
CWR_i_: Water requirement for *i* type crop
C_WTP_: Wastewater generated from commercial
D_A_: Agricultural demand
DA_inf-SA_: Inflow from a deep aquifer to SA
D_Am_: Amenities demand
D_BNPD_: Annual bulk demand of population independent sectors
D_BPD_: Annual bulk demand of population dependent sectors
D_I_: Industrial demand
D_M_: Municipal demand
D_R_: Residential demand
DW_A_: Annual Desalinated water consumption by agriculture
DW_AM_: Annual Desalinated water consumption by amenities
DW_AR-SA_: Annual Artificial recharge to shallow aquifer
DW_AR-SA_: Artificial recharge of DW
DW_C_: Annual Desalinated water consumption by commercial
DW_F_: Annual Desalinated water consumption by forestry
DW_I_: Annual Desalinated water consumption by industrial
DW_inf-SA_: Infiltration to SA from DW leakage and loss
DW_M_: Annual Desalinated water consumption by municipal
DW_R_: Annual Desalinated water consumption by residential
DW_Total_: Total annual supply from DW
F_inf-SA_: infiltration to SA from forestry
FWR_r_: Water requirement for *r* region forest
GW_A_: Annual Groundwater consumption by agriculture
GW_AM_: Annual Groundwater consumption by amenities
GWE_inf-SA_: External aquifer inflow
GW_F_: Annual Groundwater consumption by forestry
GW_Total_: The total annual supply from GW
IE_A_: Irrigation efficiency at agricultural lands
IE_Am_: Irrigation efficiency at amenities
IE_F_: Irrigation efficiency at forest lands
inf_WTP_: Infiltrated water reaching WTPs
I_WTP_: Wastewater generated from industrial
k: Type of amenities facilities
Km^2^: Square Kilo meter
lpcd: liters per capita per day
L_r_: Leaching requirement
MCM/yr: Million cubic meter per year
M_WTP_: Wastewater generated from municipal
N_cw_: Number of car wash units
N_hr_: Number of hotel room
N_hs-bed_: Number of hospital bed
Nj: Number of *j* type households
N_mq_: Number of mosques
N_off-emp_: Number of office employees
N_ret-emp_: Number of retail employees
N_st_: Number of students
N_vs_: Number of visitors to recreation places
O_hr_: Occupancy rate of hotel rooms
P: Population
PCD_Am_: Per capita amenities demand per day
PCD_C_: Per capita commercial demand per day
PCD_M_: Per capita municipal demand per day
PCD_PD_: *lpcd* determined for respective population related sectors
PCD_R_: Per capita consumption per day for residential demand
PCD_R-n_: Nationals’ per capita consumption per day for residential demand
PCD_R-nn_: Non-Nationals’ per capita consumption per day for residential demand
P_n_: Nationals’ population
P_nn_: Non-nationals’ population
PWR: Potable water return ratio
R: Regions of forestry: eastern and western
RF_SR_Total_: Total annual surface-runoff
RF_E-OA_: Evaporation components of rainfall received
RF_inf-SA_: Natural rainfall recharge
RF_inf-SA_: Rainfall infiltrated to shallow aquifer
RF_SDS_: Rainfall component reaching storm drainage system
RF_Total_: Total annual RF
R_inf-SA_: Infiltration to SA from residential outdoor use
RR_TS_: Recycle ratio of produced TS
R_WTP_: Wastewater generated from residential
SA_inf-SDS_: Infiltration of GW from SA to SDS
SA_Total-inflow_: Total recharge into the SA
SDS_Total-Inflow_: Total annual water discharge into sea through storm drainage system
TS_AM_: Annual TS consumption by amenities
TS_F_: Annual TS consumption by forestry
TS_Sea_: Annual TS discharged into sea
TS_Total_: Total annual supply from TS
TS_Total_: Total annual reusable TS produced
WS_Total_: Total annual water supply
WTP: Wastewater treatment plants
WTP_Total-inflow_: Total wastewater inflow at WTP
